# Functional analysis of a novel nonsense variant c.91A>T of the *TRAPPC2* gene in a Chinese family with X-linked recessive autosomal spondyloepiphyseal dysplasia tarda

**DOI:** 10.3389/fgene.2023.1216592

**Published:** 2023-08-25

**Authors:** Guiyu Lou, Yuanyin Zhao, Huiru Zhao, Yuwei Zhang, Bingtao Hao, Litao Qin, Hongyan Liu, Shixiu Liao

**Affiliations:** ^1^ Henan Provincial People’s Hospital, Medical Genetics Institute of Henan Province, Henan Provincial Key Laboratory of Genetic Diseases and Functional Genomics, People’s Hospital of Zhengzhou University, Zhengzhou, China; ^2^ Department of Biochemistry and Molecular Biology, College of Basic Medical Science, Army Medical University, Chongqing, China

**Keywords:** X-linked spondyloepiphyseal dysplasia tarda, TRAPPC2, COL2A1, whole-exome sequencing, nonsense variant

## Abstract

Spondyloepiphyseal dysplasia tarda (SEDT) is a condition involving late-onset, X-linked recessive skeletal dysplasia caused by mutations in the *TRAPPC2* gene. In this paper, we identified a novel nonsense variant in a SEDT pedigree and analyzed the function of the variant in an attempt to explain the new pathogenesis of the TRAPPC2 protein in SEDT. Briefly, DNA and RNA samples from the peripheral blood of SEDT individuals were prepared. The causative variant in the Chinese SEDT family was identified by clinic whole-exome sequencing analysis. Then, we observed the mRNA expression of *TRAPPC2* in patients and the mutant TRAPPC2 level *in vitro* and analyzed the protein stability and subcellular distribution by cell fluorescence and Western blotting. We also investigated the effect of *TRAPPC2* knockdown on the expression and secretion of COL2A1 in SW1353 cells or primary human chondrocytes. Herein, we found a nonsense variant, c.91A>T, of the *TRAPPC2* gene in the pedigree. *TRAPPC2* mRNA expression levels were significantly decreased in the available peripheral blood cell samples of two affected patients. An *in vitro* study showed that the mutant plasmid exhibited significantly lower mRNA and protein of *TRAPPC2*, and the mutant protein changed its membrane distribution. *TRAPPC2* knockdown resulted in decreased *COL2A1* expression and collagen II secretions. Our data indicate that the novel nonsense variant, c.91A>T, of the *TRAPPC2* gene is the cause of SEDT in this pedigree. The variant results in a lowered expression of TRAPPC2 and then affects the *COL2A1* expression and collagen II secretions, which may explain the mechanism of loss of function of the variant.

## Introduction

Spondyloepiphyseal dysplasia tarda (SEDT) is a condition involving late-onset, X-linked recessive osteochondrodysplasia with a prevalence of 1.7 individuals per million. Major clinical manifestations include disproportionate short stature, degenerative osteoarthritis, narrowing of the intervertebral disc spaces, and platyspondyly (Gedeon et al., 1999; Savarirayan et al., 2003).

The *TRAPPC2* gene was the first known causal gene of this disease, encoding a protein product named Sedlin that was involved in multiple processes of intracellular transport activities (Gedeon et al., 1999). Typical defects in the *TRAPPC2* gene were frame-shifting deletions and splicing errors, resulting in the loss of function of this gene (Gedeon et al., 2001; Tiller et al., 2001; Xia et al., 2014; Won et al., 2019). Currently, over 60 pathogenic variants are reported, expanding the defect spectrum to missense variants and nonsense variants (Stenson et al., 2017). However, only a few variants have been studied functionally. For example, the c.1 A>T or c.40del G variants altered their translation level (Won et al., 2019). The c.94-2 A>G variant resulted in the loss of exon 4 skipping, producing a truncated Sedlin protein (Fukuma et al., 2018).

At the molecular level, Sedlin can bind to other components of the trafficking protein particle (TRAPP) complex and play a critical role in the traffic of vesicles between the endoplasmic reticulum (ER) and the Golgi apparatus (Gecz et al., 2000; Sacher et al., 2001). It controlled the ER export of procollagen by regulating the Sar1 cycle (Venditti et al., 2012). Moreover, Sedlin interacted with multiple transcription factors regulating genes important in skeletal development (Ghosh et al., 2001; Fan et al., 2003).

Nonsense variants were not commonly seen in *TRAPPC2* (Choi et al., 2009; Brunet and Sacher, 2014). In this study, a novel nonsense variant c.91A>T (p.K31X) was identified in two cousins of a Chinese pedigree diagnosed with SEDT. This allowed us to investigate the molecular mechanism of the K31X variant in *TRAPPC2* within the context of SEDT pathogenesis.

## Materials and methods

### Participants and IRB approval

**FIGURE 1:**
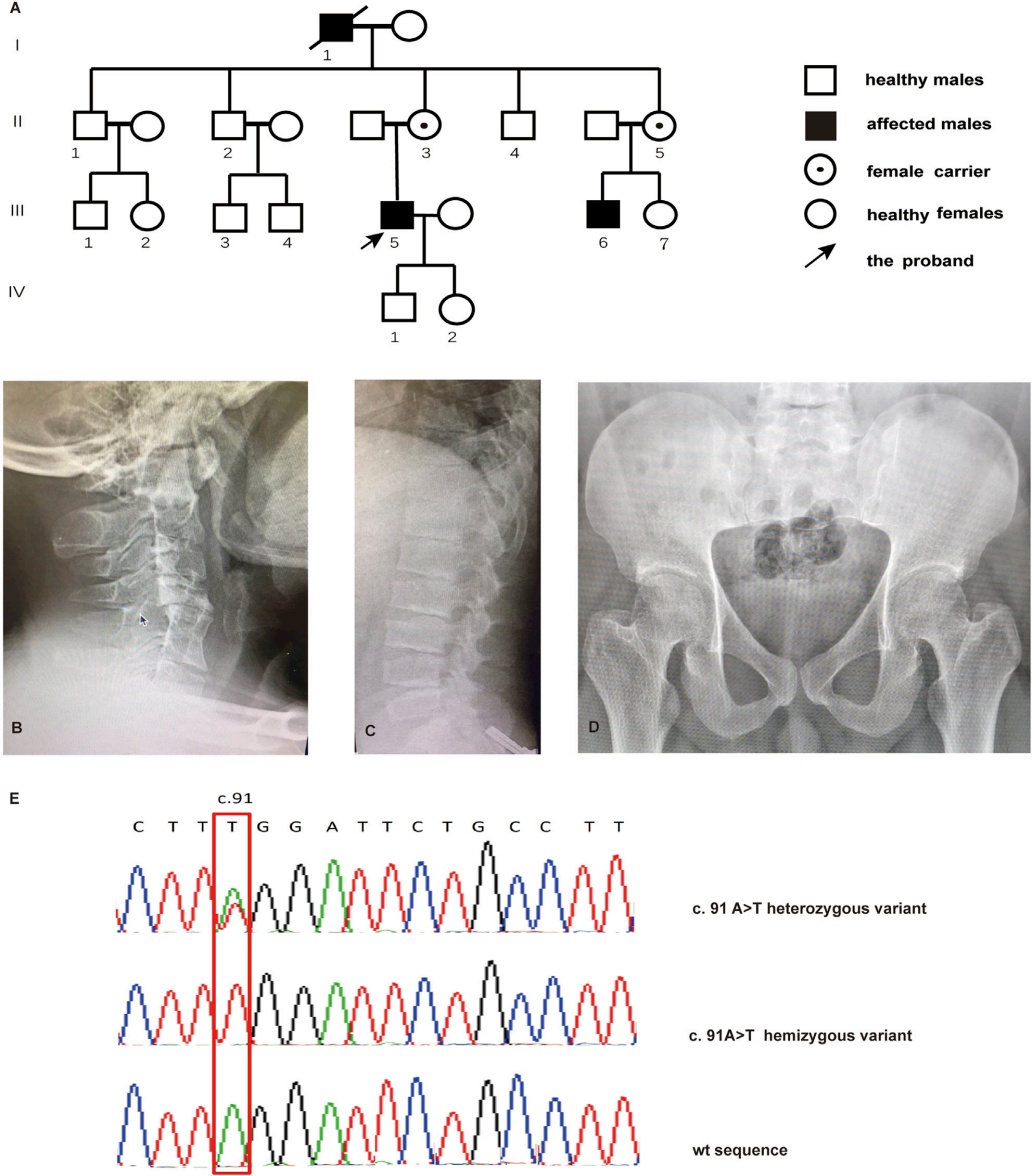
Pedigree of the family with SEDT and sequencing analysis. **(A)** Pedigree of the family. Radiographs for cervical vertebra **(B)**, lumbar vertebra **(C)**, and pelvis **(D)** of the proband with SEDT. **(E)** Sanger sequencing chromatograms indicating male individuals affected with the hemizygous TRAPPC2 c.91 A>T variant and female individuals affected with the heterozygous variant.

Two SEDT patients and their healthy family members were enrolled in this study (Figure 1A). The family members’ medical history, general physical examination, and femur and spine X-ray examination were collected. Written consent was obtained from all participants before the collection of blood samples. The study was approved by the Institutional Research Ethics Board of Henan Provincial People’s Hospital (ref. no 2021-171.)

### Whole-exome sequencing and sequence analysis

Whole-exome sequencing (WES) was performed on the proband. Exome targets were captured using the Agilent Inherited Disease Panel (Agilent Technologies, Inc.) and sequenced on the HiSeq 2500 platform (Illumina, Inc.). Read alignment against the human reference genome builds hg19 was performed using NextGENe software. INGENUITY software was used to annotate variants. The suspected variant was verified in the family numbers by Sanger sequencing.

### Plasmid construction

The full-length human *TRAPPC2* cDNA (NM_001011658) was cloned into the pEGFP-C1 vector using the In-Fusion HD Cloning kit (Takara Bio, United States) to produce a recombinant plasmid, pEGFP-TRAPPC2. Mutant plasmids with the p.K31X mutation, pEGFP-TRAPPC2-K31X, were constructed using the QuikChange Site-directed mutagenesis kit (QuikChange II Mutagenesis Kit, QIAGEN). Plasmids were sequenced to ensure that the correct sequences were produced.

### Isolation of chondrocytes from human articular cartilage

Femoral head cartilage specimens of healthy people who underwent surgeries were collected after obtaining informed consent and were then minced using a sterile scalpel into small pieces (approximately 2 × 2 mm) in a Petri dish containing PBS. According to the methods described previously (Jeyabalan, et al., 2010), PBS containing the minced cartilage was carefully removed using a pipette, and the Petri dish was immediately filled with 0.1% collagenase II (Sigma) for overnight indigestion (20 h) at 37°C with 5% CO_2_. Next, the cell suspension was dispersed through a cell strainer (70 μm; Falcon) and washed two times by centrifugation in DMEM/F-12. The cell pellet was then resuspended after the final wash in DMEM/F-12 supplemented with 10% FBS (HyClone) and 1% antibiotic–antimycotic (Corning).

### Cell culture and transfection

HEK293T cells were cultured in DMEM supplemented with 10% FBS, 100 µ/mL penicillin, and 100 µ/mL streptomycin at 37°C with 5% CO_2_. HEK293T cells were transfected with pEGFP-TRAPPC2 or pEGFP-TRAPPC2-K31X plasmids by Lipofectamine 2000 (Invitrogen). The DsRed vector was co-transfected. qPCR was performed 24 h post-transfection. Protein expression was measured by fluorescence microscopy and Western blotting 48 h post-transfection. The chondrosarcoma SW1353 cells and primary chondrocytes were cultured in DMEM/F12 supplemented with 10% FBS, 100 µ/mL penicillin, and 100 µ/mL streptomycin at 37°C with 5% CO_2_. Transient transfection of small interfering RNAs (siRNAs) targeting TRAPPC2 (5′-GGG​CAU​AUG​AGG​UUU​AUU​ATT-3′), along with the control, was performed with Lipofectamine RNAiMAX (Invitrogen) according to the manufacturer’s instructions.

### RNA extraction and quantitative reverse transcription-PCR assays

RNA was isolated from the peripheral blood mononuclear cells (PBMCs) of two affected patients, one normal male control from the affected family, and five unrelated healthy controls using the RNeasy Mini RNA Isolation Kit (QIAGEN). The total RNA from HEK293T cells and SW1353 cells post-transfection was extracted using TRIzol (Invitrogen). The RNA was treated with DNase (QIAGEN) in order to remove genomic DNA contamination before being reverse-transcribed using the RevertAid First Strand cDNA Synthesis Kit (Thermo Scientific). Quantitative real-time PCR was performed using PowerUP^TM^ SYBR Green Master Mix (Applied Biosystems). The comparative ΔΔCT method of the relative quantification strategy was used to evaluate differences in gene expression. The mRNA levels were normalized to glyceraldehyde-3-phosphate dehydrogenase (*GAPDH*) levels. Primers used for quantitative PCR analysis were designed as follows: *TRAPPC2*-F: 5′-TGC​TGT​GAG​GTA​AGG​AGC​C-3′, *TRAPPC2*-R: 5′-GTC​GAG​AGC​AGC​ATG​AGC​TA-3′; *GFP*-F: 5′-AAG​CAG​AAG​AAC​GGC​ATC​AA-3′, *GFP*-R: 5′-GGG​GGT​GTT​CTG​CTG​GTA​GT-3'; and COL2A1-F: 5′-AAC​CAG​ATT​GAG​AGC​ATC​CG-3′, *COL2A1*-R: 5′-AAC​GTT​TGC​TGG​ATT​GGG​GT-3'. *TRAPPC2B* (also known as SEDLP1) is the pseudogene of *TRAPPC2*, located at 19q13.4, with only six nucleotide differences in the ORFs of the two sequences (Gécz et al., 2000). Data from GTEx show that they are expressed in various tissues, among which the expression of *TRAPPC2B* in whole blood is approximately 20% of that of *TRAPPC2*. Therefore, it is necessary to design specific primers for *TRAPPC2* transcripts to exclude interference from *TRAPPC2* transcripts.

### Western blot analysis

Total proteins in cell lysates from HEK293T cells and SW1353 cells were isolated by RIPA extraction buffer supplemented with a protease inhibitor cocktail. The membrane and cytosol protein were extracted using the kit from Beyotime Biotechnology (China) according to the manufacturer’s instructions. The protein concentration was detected using the BCA Protein Quantification Kit (Yeasen Biotech, Shanghai, China). The protein was separated by 10% SDS-PAGE and transferred to a 0.45-µm PVDF membrane. An hour later after incubation at room temperature in blotting buffer, the membrane was incubated overnight with the primary antibody against GFP (Cell Signaling Technology, 1:1000), TRAPPC2 (Abcam, 1:1000), COL2A1 (Abcam, 1:1000), or GAPDH (Affinity, 1:1000), followed by incubation with the appropriate secondary antibodies for 1 h. After immunoblotting, an ECL Plus Western Blotting System (Yeasen Biotech, Shanghai, China) was utilized to visualize the results.

### Confocal microscopy

Cells were seeded on sterile coverslips in 24-well plates and then co-transfected with Sedlin–EGFP constructs and pDsRED-Monomer-Golgi (Takara). At 24 h post-transfection, the cells were washed with PBS and fixed with 4% formaldehyde for 5 min. After washing, the coverslips were incubated with DAPI for 5 min and mounted with ProLong Gold Mounting medium (Thermo Fisher Scientific). Immunostaining cell images were captured using Zeiss LSM Image Browser and Zen 2.1 (Carl Zeiss) and organized using ImageJ.

### Fluorescence microscopy

Transfected HEK293T cells were seeded and transfected on a six-well plate, and the expression of the pEGFP-C1-TRAPPC2 plasmid was observed by fluorescence microscopy under 475 nm excitation light.

### Detection of secreted collagen II from the cell culture medium

Primary chondrocytes transfected with *TRAPPC2* siRNA were cultured in six-well plates with 10% FBS DMEM at 37°C with 5% CO_2_. The culture medium was collected 24 h post-transfection. COL2A1 was detected according to the enzyme-linked immunosorbent assay (ELISA) kit’s protocol (Elabscience).

### Statistical analysis

Group differences were tested by Student’s t-test or two-way ANOVA using GraphPad Prism (GraphPad Software).

## Results

### Clinical presentations and sequencing analysis

The four-generation pedigree is consistent with X-linked recessive inheritance according to the inherent characteristics that patients are male and patient mothers are carriers (Figure 1A). Affected members presented short stature (III-5:140 cm; III-6:135 cm), growth retardation at a young age, abnormal walking posture, and pain in the back and large joints. Radiographs revealed cervical intervertebral disc space stenosis with adjacent vertebral sclerosis, lumbosacral platyspondyly with characteristic superior and inferior humping of the vertebral bodies observed on lateral view, and flat femoral head and femoral neck (Figures 1B–D).

WES analysis identified a novel nonsense variant c.91A>T in the *TRAPPC2* exon 3 in the proband, causing translation to terminate after the 31 amino acid (aa). Therefore, this mutation produced a truncated protein that lost 109 amino acids from the C terminal. Sanger sequencing verified the hemizygote variant in his cousin (III-6). II-3 and II-5 were found to be heterozygous for the c.91A>T substitution, confirming their carrier status (Figure 1E). Based on the ACMG criteria, the novel c.91A>T variant in the *TRAPPC2* gene was the pathogenic variant of this X-linked SEDT family.

### Impact of K31X on *TRAPPC2* gene expression *in vivo* and *in vitro*

**FIGURE 2:**
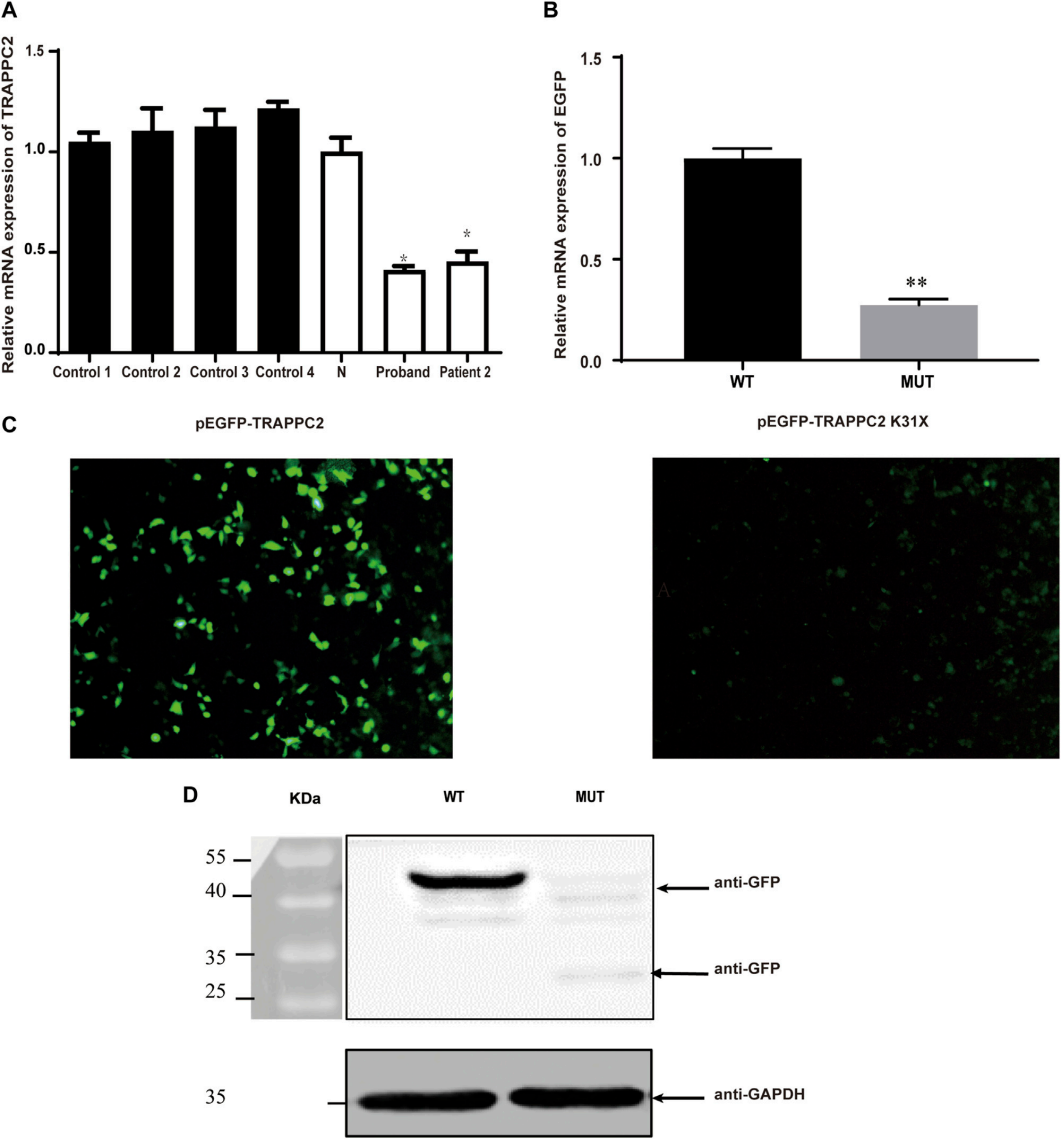
Effects of c.91A>T (K31X) on TRAPPC2 expression. **(A)** TRAPPC2 mRNA expression in PBMCs. *p < 0.05 compared with the N or control group (*t*-test) and the results of qRT-PCR for the mRNA levels of EGFP in 293T cells. **Significantly different from the wild-type plasmid (*t*-test, *p* < 0.01). Controls 1, 2, 3, and 4: healthy controls from an unrelated population; N: healthy control in the pedigree. HEK293T cells were transfected with 2 μg wild-type pEGFP-C1-TRAPPC2 or mutant plasmids (pEGFP-C1-TRAPPC2; pEGFP-TRAPPC2-K31X) with 5 μL Lipofectamine 2000. EGFP mRNA levels were detected by qRT-PCR **(B)**. It showed a lower level of EGFP protein expression observed by fluorescence microscopy **(C)**. Scale bar: 100 µm (×20 magnification). **(D)** Immunoblotting using antibodies against GFP showed EGFP-labeled TRAPPC2 expression, with a reduction in the mutant product (∼43 kDa for WT; ∼30 kDa for mutant) in 293T cells.

Real-time PCR showed that the expression of mutant *TRAPPC2* in PBMCs was decreased by ∼52% compared to healthy controls (Figure 2A). Transfection of HEK293T cells by the pEGFP-C1-TRAPPC2-K31X plasmid also showed a decreased Sedlin expression level (Figure 2B) 24 h post-transfection and even more decreased expression level 48 h post-transfection (Figure 2C). Furthermore, Western blotting was performed to confirm the reduced expression of the protein with premature truncation K31X (Figure 2D).

### Disrupted subcellular localization of the K31X Sedlin protein

**FIGURE 3:**
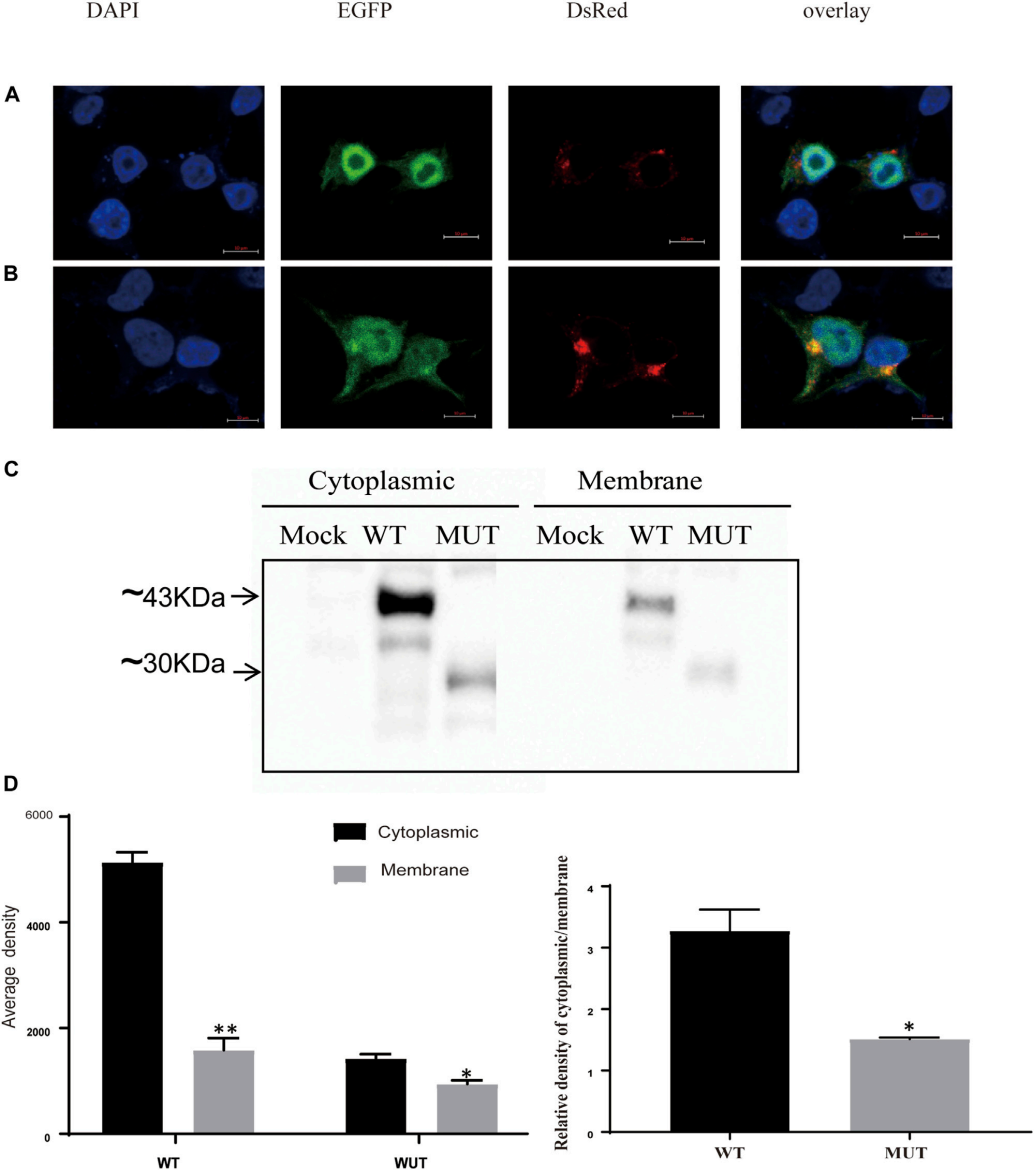
Subcellular localization of Sedlin and impact of truncation. Confocal microscopy of WT Sedlin **(A)** and mutant Sedlin **(B)** localization in HEK293T cells (co-transfected with 1.5 μg EGFP-tagged WT or mutant Sedlin and a 0.5 μg pDsRED-Monomer-Golgi vector). Golgi apparatus: red; nucleus: blue; TRAPPC2: green. **(C)** Western blot results for cytoplasm and membrane fractions. Mock: HEK293T cells without transfection; WT: HEK293T cells transfected with pEGFP-C1-TRAPPC2; MUT: HEK293T cells transfected with pEGFP-TRAPPC2-K31X; primary antibody: anti-EGFP. **(D)** Quantified signal intensities of Western blotting. *p < 0.05, **p < 0.01, membrane compared with cytoplasmic fractions, or *p < 0.05 MUT group compared with the WT group by Student’s t-test.

We next investigated the subcellular localization of the Sedlin protein, as this has been known to be important for its cellular functions (Choi et al., 2009; Jeyabalan et al., 2010). In 293T cells, wild-type Sedlin was expressed in the nucleus, granular structures enclosing the nucleus, and the Golgi apparatus (Figure 3A). Although the mutant protein was still present enclosing the nucleus and in the Golgi apparatus, there was an increase in the Golgi (Figure 3B). Furthermore, to quantify the distribution, the cytoplasm and membrane components of the same sample were collected through gradient fractionation for Western blotting. The results showed the distribution of WT and MUT Sedlin in both soluble cytoplasmic and membrane fractions (Figure 3C). The mutant Sedlin showed lower protein levels in both the cytoplasmic and membrane fractions than WT Sedlin. However, the distribution ratio of the cytoplasm to the cell membrane of the MUT group was lower than that of WT (Figure 3D), which may result from the increased distribution in Golgi.

### Mediation of *COL2A1* expression by the *TRAPPC2* gene

**FIGURE 4:**
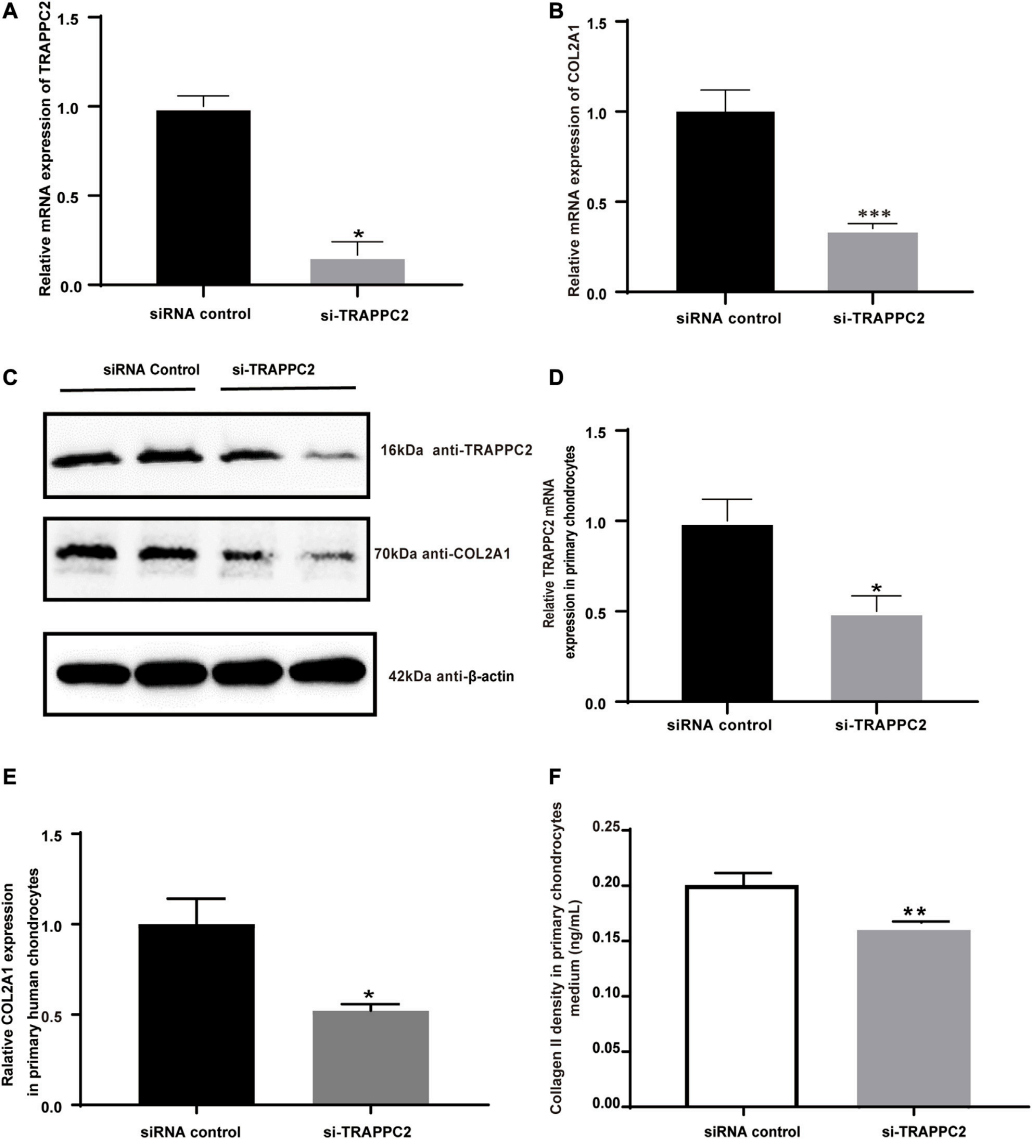
Effects of TRAPPC2 knockdown on COL2A1 expression and collagen II secretion. **(A)** TRAPPC2 and **(B)** COL2A1 transcriptional expression by siRNA of TRAPPC2 in SW1353 cells. **(C)** Western blot results of TRAPPC2 and COL2A1 expression in TRAPPC2-knockdown-SW1353 cells. **(D)** TRAPPC2 and **(E)** COL2A1 transcriptional expression by siRNA of TRAPPC2 in primary human chondrocytes. **(F)** Changes in collagen II secretion after 24 h post-transfection in primary human chondrocytes after siRNA transfection, detected by ELISA. *p < 0.05, **p < 0.01, ***p < 0.001 compared with the siRNA control group by Student’s t-test.

*COL2A1* encoded a component of type II collagen, which was abundantly present in cartilage. Given its critical role in cartilage and bone development, its relation with *TRAPPC2* was investigated. Here, siRNAs were used to knock down *TRAPPC2* expression in the chondrosarcoma cell line SW1353 and primary chondrocytes. The efficacy of knockdown is shown in Figures 4A, D. Because TRAPPC2B has the potential to produce a protein identical to that coded by TRAPPC2, it is difficult for the antibody to distinguish the two proteins. The real TRAPPC2 protein expression after knockdown may be interfered with by the presence of TRAPPC2B. In conclusion, qPCR and Western blotting showed decreased *COL2A1* expression in response to *TRAPPC2* knockdown (Figures 4B, C) in SW1353 cells. Further work was performed in primary chondrocytes, and decreased *COL2A1* mRNA level and collagen Ⅱ secretion was observed in the *TRAPPC2* knockdown group, supporting the role of *TRAPPC2* in mediating *COL2A1* expression (Figures 4E, F).

## Discussion

A pedigree that includes four generations and three male patients with X-linked recessive inheritance was described. According to the clinical manifestations and typical X-rays, the clinical diagnosis of X-linked SEDT can be established in the pedigree. The molecular genetic testing of X-linked SEDT in male patients found a novel hemizygous nonsense c.91A>T variant in *TRAPPC2* (NM_001011658)17. Currently, a total of nine nonsense mutations have been reported. However, a characterization of the effects of the nonsense mutations in *TRAPPC2* was still lacking. The present work focused on the possible mechanisms in patients bearing nonsense c.91A>T variant mutation.

This study focused on the reported truncating mutation in *TRAPPC2* at codon 31 and explored its molecular mechanism underlying the disease. We investigated molecular mechanisms involving the regulation of *TRAPPC2* gene expression both at mRNA and protein levels, along with its subcellular location changes and downstream targets. Our data suggested that truncation of Sedlin, although not observed often in SEDT patients, represented an important mechanism in the loss of function of this gene. Crystal structures indicated Sedlin to be a monomer of mixed α/β-fold structures made up of a central β-sheet (Jang et al., 2002). The domain structure contained 20 solvent-accessible apolar residues. Four of these apolar residues constituted a hydrophobic pocket (Pro16) and a hydrophobic groove (Phe40, Leu48, and Phe67), which could interact with c-Myc promoter-binding protein 1 (MBP1), pituitary homeobox 1 (PITX1), and steroidogenic factor 1 (SF1), proteins important in endochondral ossification and maintenance of adult bones (Jeyabalan et al., 2010). The mechanisms that transport Sedlin into the nucleus remain to be defined. However, like other studies, our experiment results also showed a Sedlin-localized nucleus. We speculate that the reduced aggregates of Sedlin in the nucleus or changes in cellular localization may interfere in the interactions between Sedlin and these transcription factors, which could be the mechanism of the downregulation of the *COL2A1* expression level. Sedlin was known to be required for transporting procollagens from the endoplasmic reticulum to the Golgi apparatus (Venditti et al., 2012). Our result indicated the role of *TRAPPC2* in the regulation of *COL2A1* gene expression, which perhaps helps explain the pathological basis for SEDT from another perspective. Indeed, whether Sedlin interacts with other transcriptional factors must be further elucidated.

## Conclusion

The novel nonsense variant c.91A>T (p.K31X) of *TRAPPC2* is pathogenic, which reduced the expression of the *TRAPPC2* gene both at the mRNA level and protein level, disrupted its protein subcellular localization, and further impacted the expression of protein COL2A1. Truncation of *TRAPPC2* represented an important molecular mechanism underlying the pathogenesis of SEDT and highlighted the importance of molecular testing in the diagnosis of this rare disease in clinics.

## Data Availability

Data deposited in https://www.ncbi.nlm.nih.gov/clinvar/, accession number SCV004023247.
